# Suicide Risk and Help-Seeking Behavior Among United States Collegiate Student-Athletes

**DOI:** 10.3390/bs16081314

**Published:** 2026-08-03

**Authors:** Allison E. Bond, Samuel V. Gerry, Taylor R. Rodriguez, Jessica L. Hamilton

**Affiliations:** 1 The New Jersey Gun Violence Research Center, Rutgers School of Public Health, Rutgers University, New Brunswick, NJ 08901, USA; 2 Department of Psychology, Bates College, Lewiston, ME 04240, USA; 3 The Department of Psychology, The University of Mississippi, Oxford, MS 38677, USA; 4 Department of Psychology, Rutgers University, New Brunswick, NJ 08901, USA

**Keywords:** suicide, student athletes, college, help-seeking, prevention

## Abstract

Objective. This study examined suicidal ideation, preferred help-seeking sources, and knowledge of prevention strategies among collegiate athletes, and whether training influences stigma or knowledge. Methods. Collegiate athletes (*N* = 78), aged 18+ and participating in NCAA DI, DII, or DIII sports, were recruited via social media and email. Participants completed self-report measures on suicide, prevention training, and help-seeking. Results. A total of 39.7% reported lifetime suicidal ideation, yet only 35.8% had received suicide prevention training in college. Teammates (46.9%) and parents/guardians (40.7%) were the most commonly endorsed sources of support. Those who received training did not differ in stigma levels or knowledge of supportive actions compared to untrained peers. Conclusions. Collegiate athletes report high rates of suicidal ideation but limited access to effective prevention training. Moreover, current training may not improve stigma or knowledge. Efforts should focus on improving and expanding suicide prevention education within athletic programs.

## 1. Introduction

In the United States, suicide is a pressing concern among college students (Centers for Disease Control and Prevention, 2023; Turner et al., 2013). Specifically, suicide is the third leading cause of death among this age group (18–22 years old) and accounts for over 3000 deaths annually (Centers for Disease Control and Prevention, 2023). College students are a heterogenous group, and therefore some subgroups may be at increased risk for experiencing suicidal thoughts and behaviors. Given this, it is critical to better understand suicide risk among different groups of college students to increase prevention efforts on campuses and ultimately reduce suicide rates. One such group is collegiate student-athletes.^1^ In recent years, several collegiate student-athlete suicide deaths have garnered national attention. The death of Madison Holleran, a runner at the University of Pennsylvania, and Katie Meyer, a soccer goalie at Stanford University, received media coverage (Fagan, 2015; McCarriston, 2022) and led to professional athletes discussing mental health across media platforms and in press interviews (e.g., Bregman, 2023; Kliegman, 2022; Stone, 2022). These and the deaths of many other National Collegiate Athletic Association (NCAA) athletes (Hensley-Clancy, 2022) highlight the pressing problem of suicide among collegiate student-athletes.

Despite media attention, limited research has examined if collegiate student-athletes are at increased risk for suicide compared to their non-athlete peers. The research that does exist indicates that student-athletes report lower rates of suicide than their peers. For example, in a sample of 165,210 undergraduates, researchers found that student-athletes were significantly less likely to think about suicide compared to their non-athlete peers (Anchuri et al., 2020). Joiner’s (2005) Interpersonal-Psychological Theory (IPT) of Suicide posits that those who perceive themselves as a burden to others (i.e., perceived burdensomeness) and feel as though they lack social cohesion (i.e., thwarted belongingness) may be at increased risk for developing suicidal ideation.

As such, student-athletes’ increased levels of social connectedness and self-esteem (Armstrong & Oomen-Early, 2009) may serve as protection against the development of suicidal thoughts. Student-athletes have been shown to endorse higher levels of fearlessness about death and subjective/objective pain tolerance—which are associated with higher capability for suicide—when compared to non-athletes (Dodd et al., 2021). According to the IPT, capability for suicide is developed through repetitive exposure to events that are painful and provocative. Sports-related injuries and physical contact in sports are conceptualized as painful and provocative events that may increase one’s capability for suicide (Dodd et al., 2021). This is concerning given that capability for suicide is the mechanism that moves an individual from thinking about suicide to attempting suicide. Therefore, while student-athletes have previously reported lower rates of suicidal ideation compared to their peers, when they do develop thoughts of suicide, they may be more able to act on them and ultimately attempt suicide.

Student-athletes also have unique experiences including performance pressure, injury, and loss of identity when exiting the sport. These unique stressors may increase their vulnerability to experiencing mental health difficulties and suicide risk. Fletcher et al. (2012) conceptualized stress in sport as arising from multiple domains, including organizational (e.g., scholarship concerns, balancing academic and athletic responsibilities), performance (e.g., performance expectations, pressure to succeed, injury, and playing time), and personal (e.g., identity development, interpersonal relationships, and life transitions). Collegiate student-athletes navigate these stressors while also managing the challenges associated with college and emerging adulthood. Furthermore, athletic identity, which is conceptualized as the degree to which an individual defines themselves through sport participation, may increase vulnerability during periods of injury or retirement; they may feel isolated and experience low self-worth or a lack of purpose, which may be associated with poorer mental health outcomes.

Given this potential risk, it is critical that student-athletes are aware of available resources and feel their mental health is supported by their respective institutions. However, little is known about collegiate student-athletes’ knowledge and use of mental health resources. In a qualitative study seeking to better understand NCAA Division I athletes’ (*N* = 23) experiences with mental health resources, many participants knew of on-campus resources (i.e., counseling) that were available to them (Young et al., 2022). Even if student-athletes are informed of mental health services and resources on their respective campuses, efforts must be made to ensure that these students perceive these resources as accessible. In a study examining mental health service utilization and help-seeking practices among collegiate student-athletes in the United States, Harris and Maher (2022) found that, despite facing mental health challenges, participants felt that they were unable to seek help due to stigma within teams, concerns about coach reception, the hours and locations of services, and poor previous experiences with said services.

One way to decrease stigma around help-seeking is to provide education to student-athletes, coaches, and athletic department staff. Previous research has found that suicide prevention trainings increase knowledge about suicide and self-efficacy to discuss suicide (Zinzow et al., 2020). Trainings can positively influence the beliefs and behaviors of those who receive them. However, the effects of suicide prevention trainings on student-athletes and their coaches have yet to be investigated, and few, if any, trainings have been catered to student-athletes, thus missing out on a potentially at-risk subgroup on college and university campuses.

To fill these gaps, the present study seeks to examine suicide risk, suicide-related knowledge, and preferred sources of help-seeking among collegiate student-athletes. In this study, suicide-related knowledge refers to participants’ understanding of suicide warning signs, risk factors, and responses to individuals experiencing suicidal thoughts. Furthermore, the study explores the extent and impact of suicide prevention training received on perceptions of suicide stigma and their readiness to respond to a teammate in crisis. Finally, we explore if there are significant differences between student-athletes who have received mental health or suicide prevention training and those who have not, in terms of stigma towards suicide. Given the exploratory nature of this study, no hypotheses are generated. Findings from this study will provide insights into suicide risk among student-athletes and can help to inform the development of targeted interventions to support their well-being.

## 2. Method

### 2.1. Procedures

The study was approved by the relevant university Institutional Review Board, and written consent was obtained from all participants. Data were collected from May 2022 to October 2023. Participation was voluntary, and participants could withdraw at any point during the survey. Participants were recruited by distributing a study flier on social media websites and emailing NCAA coaches and athletic directors a survey link to be forwarded to prospective participants. Participants were recruited through advertisements posted on X (formerly Twitter) and Instagram. Additionally, we reached out to over 20 coaches and athletic directors and asked them to share with their network as well. The survey was anonymous, and no personally identifying information was included. Participants completed the survey on Qualtrics, and to protect participants’ anonymity, their IP address, which was collected via Qualtrics, was not connected to their data.

Participants completed a brief, five-minute survey, and no compensation was provided. Inclusion criteria included being 18 years of age or older and a current student-athlete at an NCAA Division I, II, or III college or university.

### 2.2. Measures

#### 2.2.1. Suicidal Ideation

Lifetime suicidal ideation was assessed via the self-report version of the Self-Injurious Thoughts and Behaviors Interview-Revised (SITBI-R; Fox et al., 2020). Participants indicated whether they had ever experienced any of eight specific types of suicidal thought: I wish I could disappear or not exist; I wish I was never born; My life is not worth living; I wish I could go to sleep and never wake up; I wish I were dead; Maybe I should kill myself; I should kill myself; and I am going to kill myself. Endorsement of one or more of these items resulted in being coded as having lifetime suicidal ideation. The scale demonstrates good internal consistency (α = 0.873).

#### 2.2.2. Help-Seeking

Items assessing preferred help-seeking sources were adapted from the General Help-Seeking Questionnaire (GHSQ; Wilson et al., 2005). To ensure its relevance to the student-athlete population, additional sources such as teammates and coaches were added to the measure. Participants were presented with the following prompt, “If you had thoughts of suicide, would you reach out to any of the following sources? Please select all that apply.” Response options included: Teammates, coaches, athletic trainers, other athletic staff, parents/guardians, psychologist, psychiatrist, student counseling center, and suicide prevention lifeline.

#### 2.2.3. Stigma

The Stigma of Suicide Scale-Short Form (SOSS-SF; Batterham et al., 2013) was used to measure stigmatization towards suicide decedents. Participants were asked to rate how much they agree with 16 one-word descriptors across three categories (i.e., Stigma, Isolation/Depression, and Glorification/Normalization), with responses of 1 (Strongly Disagree) to 5 (Strongly Agree). Higher scores indicate higher levels of stigma. The scale demonstrates good internal consistency (α = 0.930).

#### 2.2.4. Suicide Prevention Training

Exposure to suicide prevention training was measured using a single item, “Have you received training on suicide prevention while in college?” Response options allowed participants to indicate whether they had received training through their athletic department and/or their college or university. Similarly, attitude towards NCAA-mandated training was measured using a single item, “Do you believe the NCAA should require all athletes to learn about mental health and suicide prevention?” Response options included yes, no, or unsure. This measure was developed by the research team because existing measures of suicide prevention training did not specifically assess training experiences among college student-athletes or within athletic settings. The measure consisted of a single-item question assessing participants’ prior exposure to suicide prevention training and their attitudes toward NCAA training requirements. Formal psychometric evaluation was not conducted. Item development was informed by relevant literature and consultation with a leading expert in youth suicide prevention to ensure content relevance and applicability to the target population.

### 2.3. Analyses

A priori power analyses were not conducted. For the regression, a power analysis indicated that the study was powered (α = 0.05, power = 0.80) to detect relatively large effects. This suggests that smaller effects may not have been detectable in the present sample. For the ANOVA, post hoc power analysis indicated that the study was powered (α = 0.05, power = 0.80) to detect effects of approximately f = 0.32 or larger. This corresponds to a medium-to-large effect size, suggesting that smaller effects may not have been detectable in the present sample.

Descriptive statistics, including frequencies, were used to examine the prevalence of suicidal ideation among student-athletes and their help-seeking behaviors. A binary logistic regression was used to examine differences in suicide stigma among those with and without previous suicide prevention training. Those who reported receiving training through their athletic department and/or college or university were combined into a single “yes”. All analyses were conducted in SPSS 28.0. Missing data for demographic variables are reported in Table 1. For the remaining analyses, only participants who responded to all relevant questions were included. Statistical significance was set at *p* < 0.05.

## 3. Results

### 3.1. Participant Characteristics

Participants included NCAA Division I, II, and III student-athletes. The sample was predominantly female (*n* = 57) and White (*n* = 77). Sample characteristics are available in Table 1. Additional sample characteristics can be found in Table 1.

### 3.2. Suicidal Ideation and Help Seeking

In the total sample (*N* = 78), 39.7% of student-athletes reported lifetime suicidal ideation. Among those who reported lifetime ideation, (*n* = 18) 58.1% experienced suicidal ideation during college. A high percentage (*n* = 39, 63.9%) of student-athletes reported feeling comfortable and knowing what to do if a teammate told them they were having suicidal ideation.

As can be seen in Table 2, less than half of the student-athletes reported that they would reach out to their teammates (*n* = 37, 47.4%) or parents/guardians (*n* = 33, 42.3%) if they were experiencing thoughts of suicide. Other sources that participants endorsed included psychologists (*n* = 24, 30.8%), student counseling center (*n* = 16, 20.5%), suicide prevention lifeline (*n* = 17, 21.8%), coaches (*n* = 14, 17.9%), psychiatrists (*n* = 12, 15.4%), athletic trainers (*n* = 9, 11.5%), and other athletic staff (*n* = 6, 7.4%).

### 3.3. Suicide Prevention Training

Overall, (*n* = 29, 37.2%) of student-athletes reported receiving suicide prevention training during college. Of those who received training, (*n* = 24) 82.8% of the training occurred in the athletic department, and (*n* = 12) 41.4% occurred through the college/university. Of note, seven individuals received training from both the athletic department and from the college/university. Overall, 86.4% of student-athletes believe the NCAA should require all athletes to learn about mental health and suicide prevention.

### 3.4. Exploratory Analyses Examining Differences Between Those with and Without Training

As can be seen in Table 3, those who received mental health or suicide prevention training did not significantly differ in terms of their perception of stigma toward suicide decedents (OR = 1.013 [0.932, 1.001]).

## 4. Discussion

The present study examined rates of suicidal ideation, help-seeking behaviors, and the impact of suicide prevention training among NCAA collegiate student-athletes. Results indicated that 39.7% of student-athletes had experienced suicidal thoughts throughout their life, with teammates and family most frequently cited as preferred sources of support. However, most student-athletes had not received suicide prevention training, and even those who had showed no significant difference in stigma compared to those without training. These findings highlight the need for improved suicide prevention efforts tailored to collegiate student-athletes and can inform the development of targeted suicide prevention training programs for NCAA collegiate student-athletes.

While previous research has indicated that student-athletes have several factors that may protect against the development of suicidal ideation (e.g., social connectedness) (Armstrong & Oomen-Early, 2009), this study found a high prevalence of lifetime suicidal ideation among student-athletes (39.7%), notably higher than among the general collegiate population (22.3%; Mortier et al., 2018). This elevated risk is especially concerning given that student-athletes may experience more painful and provocative events, which could increase their capability for suicide (Forrest et al., 2019) and make them more at risk for attempting suicide in the future. This highlights the need for the development of suicide prevention strategies that address the unique experiences of student-athletes. However, it is important to note that previous research has indicated that participation in collegiate athletics may serve as a protective factor against suicide risk. This discrepancy between previous research and current findings may be attributable, in part, to the study’s modest sample size and potential sampling bias, which may have limited our ability to detect associations consistent with those reported in larger and more representative samples.

Findings indicate that collegiate student-athletes prefer informal help-seeking sources, such as teammates and parents, followed by more formal sources like psychologists and the student counseling center. While this finding should be interpreted with caution, given the modest sample size, it is in line with existing literature that suggests that individuals are more likely to disclose suicidal ideation to personal connections rather than professionals (e.g., Rodriguez et al., 2024). Given this, it may be important to equip student-athletes with resources and referrals for mental health care to assist their teammates in times of crisis. Additionally, the preference for parents as a help-seeking source highlights the importance of providing parents of collegiate student-athletes with appropriate resources to support their children while at college. Ensuring that student-athletes are aware of available local mental health care options and on-campus resources is vital, as approximately a quarter of them report a preference for seeking help from more professional sources. Student-athletes report preferring to seek help from coaches, athletic trainers, or other athletic staff less than other sources. While we were not able to determine the reasons for this, it may be because of concerns that disclosing suicidal ideation could negatively affect their playing time, scholarships, or other significant aspects of their athletic or academic career. It is also important to consider that coaches operate under organizational and performance-related stress themselves and are not clinically trained to navigate suicidal crisis among their athletes. In addition, coaches report experiencing isolation, a lack of support, and difficult work–life balance, which impact their mental health and burnout (McLoughlin et al., 2026). Therefore, athletic departments and institutions broadly play an important role in supporting their athletes and coaches. Departments may consider focusing on reducing stigma, and efforts should be made to educate both student-athletes and their parents about how to respond to disclosures of suicidal ideation. As mentioned above, the findings surrounding help-seeking should be interpreted with caution given the sample size, and future research is needed to validate these results.

Even though teammates are the most preferred source to seek help from, most student-athletes have not received suicide prevention training. Specifically, only 35.8% of collegiate student-athletes had received suicide prevention training during college. Among participants who reported receiving suicide prevention training, 82.8% (*n* = 24) indicated that the training was provided through their athletic department, while 41.4% (*n* = 12) reported receiving training through their college or university.

About a third of the sample received suicide prevention training; however, these participants did not differ from those without training in suicide stigma. It is possible that student-athletes experienced limited or poorly tailored training, highlighting a need for more comprehensive approaches. In line with this, we did not assess the type of training that was received, and therefore the group of student-athletes who received training may be heterogeneous. Specifically, some may have received a standardized suicide prevention training (e.g., Applied Suicide Intervention Skills Training [ASIST]), others Mental Health First Aid, and others informal training or attended talks. Gatekeeper training aims not only to de-stigmatize suicide but also to equip individuals with the tools and confidence to intervene. For example, the Question, Persuade, and Refer (QPR) Institute’s training for student-athletes aims to increase knowledge, self-efficacy, and intent to intervene. The lack of difference in attitudes and actions between trained and untrained participants, coupled with low training rates, underscores the urgent need for evidence-based suicide prevention training for student-athletes. These efforts should address the unique needs and experiences of college student-athletes and ensure adequate dosage and quality to drive meaningful changes in attitudes and behaviors.

As previously mentioned, training rarely occurs, and even when it does, it is not significantly impacting stigma or the actions one would take to assist a teammate. However, 63.9% of participants stated they know what to do if someone discloses suicidal thoughts. This finding suggests that student-athletes may feel more confident in their ability to reduce suicide risk among their peers than their reported responses demonstrate. This further highlights the need for suicide prevention training in order to ensure that student-athletes are provided with information on the appropriate steps to take when supporting a teammate experiencing thoughts of suicide.

### 4.1. Policy Considerations

The NCAA serves an important role in ensuring student-athletes receive the tools they need to support themselves and their teammates. Currently, while the NCAA requires training on mental health for coaches and administrators, it does not require any information to be provided to student-athletes. In our study, the overwhelming majority of student-athletes (86.4%) reported the NCAA should require all athletes to learn about mental health and suicide prevention. Student-athletes want this information and are currently under-equipped to help themselves and teammates during a time of crisis. In 2014, the NCAA developed the Innovations in Research and Practice Grant Program, with the goal of supporting research focused on student-athletes’ mental health, which has since been discontinued. While this was an important step in advancing mental health care among collegiate student-athletes, the gap between research and practice needs to be addressed.

### 4.2. Limitations and Future Directions

While informative, the present study is not without limitations. First, participants were recruited through email and social media postings, which may limit the representativeness of the sample relative to the broader NCAA student-athlete population. This recruitment strategy may also have introduced self-selection bias, as individuals with personal experiences related to suicide risk may have been more likely to participate. Future research should consider alternative recruitment approaches to minimize this potential bias.

Second, the sample size was modest, limiting the generalizability of findings and potentially reducing statistical power to detect meaningful effects. The small sample size also limited our ability to examine differences across racial and ethnic groups. To facilitate analyses, race was condensed into White versus non-White categories, restricting our ability to understand potential racial and ethnic differences in suicide prevention training, help-seeking behaviors, and suicidal ideation among collegiate student-athletes. Future studies should recruit larger and more diverse samples to better examine these important differences.

Third, although there was no financial incentive to participate and the data were reviewed following collection, the survey did not include a bot-detection procedure. As a result, it is possible that automated responses were included in the dataset.

Finally, several measurement limitations should be noted. The study did not assess the specific type of suicide prevention training participants received, limiting our ability to evaluate whether different training approaches were associated with distinct outcomes. Similarly, we did not assess for suicide prevention training received earlier on (e.g., middle school or high school) in one’s academic career. In addition, the assessment of help-seeking preferences did not include intimate partners or other peer-level sources, such as non-student-athlete friends and siblings. Future research should examine a broader range of help-seeking sources and explore how different types of suicide prevention training influence knowledge, attitudes, and behaviors among student-athletes.

### 4.3. Conclusions

The present study found that a high percentage of collegiate student-athletes experience suicidal ideation and are therefore at risk for suicide. While many athletes have received suicide prevention training, this study was not able to determine the types of training received. Evidence-informed suicide prevention training specifically designed to meet the unique experiences and needs of collegiate student-athletes should be developed and disseminated in partnership with the NCAA in an effort to reduce the suicide rate among student-athletes.

## Figures and Tables

**Table 1:** Sample characteristics.

Variable			
Age	M(SD)	19.34 (1.28)	
Gender		*N*	%
	Women	54	69.2%
	Men	24	30.8%
Race/Ethnicity *			
	White	74	94.9%
	Other	10	12.9%
Collegiate year	Freshman	25	35.2%
	Sophomore	18	22.1%
	Junior	17	21.8%
	Senior	9	11.5%
	Fifth Year+	2	2.6%
	Missing	7	9.0%
Seasons played at collegiate level	1	36	46.2%
	2	17	21.8%
	3	11	14.1%
	4	6	7.7%
	Missing	8	10.3%
Division			
	DI	3	3.8%
	DII	3	3.8%
	DIII	65	83.3%
	Missing	7	9.0%
Lifetime Suicidal Ideation			
	No	47	60.3%
	Yes	31	39.7%

* Participants could select multiple races or ethnicities, so percentages do not add up to 100%.

**Table 2:** Help-seeking sources.

Source	*N*	%
Teammates	37	47.4%
Parents/Guardians	33	42.3%
Psychologist	24	30.8%
Student Counseling Center	16	20.5%
Suicide Prevention Lifeline	17	21.8%
Coaches	14	17.9%
Psychiatrist	12	15.4%
Athletic Trainers	9	11.5%
Other Athletic Staff	6	7.7%

**Table 3:** Logistic regression comparing stigma among those with and without training.

	*p*	OR	CI
Stigma	0.764	1.013	0.932, 1.101

## Data Availability

Data may be made available upon reasonable request to the author.

## Note

1 Student-athletes discussed throughout this paper refer only to collegiate student-athletes.
